# Chlorogenic Acid Protects Against Indomethacin-Induced Inflammation and Mucosa Damage by Decreasing *Bacteroides*-Derived LPS

**DOI:** 10.3389/fimmu.2020.01125

**Published:** 2020-06-03

**Authors:** Yongwang Yan, Xu Zhou, Kangxiao Guo, Feng Zhou, Hongqi Yang

**Affiliations:** ^1^College of Bioscience and Biotechnology, Hunan Agricultural University, Changsha, China; ^2^Pharmaceutical College, Changsha Health Vocational College, Changsha, China; ^3^Department of Gastroenterology, Changsha Hospital of Traditional Chinese Medicine, Changsha, China

**Keywords:** chlorogenic acid, indomethacin, inflammatory bowel disease, intestinal integrity, microbiota

## Abstract

**Background:** Chlorogenic acid (CGA), a natural bioactive polyphenol, exerts anti-inflammatory, antioxidant, and antibacterial effects that support the maintenance of intestinal health. However, the influence of CGA on gut microbiota and their metabolites, as well as its potential effects and mechanism of action in inflammatory bowel disease, remain to be elucidated.

**Methods:** First, an oral gavage was used to administer CGA to indomethacin-treated mice. Then, fecal microbiota transplantation was performed to explore the role of intestinal microbiota in indomethacin-induced inflammation.

**Results:** CGA treatment protected against body weight loss, damage to intestinal morphology and integrity, inflammation, and alteration of microbiota composition in indomethacin-treated mice. Interestingly, CGA failed to inhibit inflammation or protect intestine integrity in mice treated with antibiotics. Notably, mice who had been colonized with intestinal microbiota from CGA-treated or CGA-and-indomethacin-treated mice, through the fecal microbiota transplantation program, were protected from indomethacin-induced inflammation, growth of *Bacteroides*, and the accumulation of *Bacteroides*-derived LPS, in congruence with those who had been treated with CGA.

**Conclusion:** The results suggest that CGA may protect intestine integrity and alleviate inflammatory responses, primarily by inhibiting the growth of *Bacteroides* and the accumulation of *Bacteroides*-derived LPS, in indomethacin-induced colitis. This newly identified mechanism broadens our knowledge of how CGA exerts protective effects on intestinal inflammation and provides strategies for the prevention of gastrointestinal mucosal damage in patients treated with indomethacin.

## Introduction

Chlorogenic acid (CGA), one of the most common and abundant polyphenols, is present in a wide variety of beverages and foods. This natural bioactive substance has been attracting the attention of researchers due to its strong anti-inflammatory and anti-oxidant properties, which are hypothesized to contribute to CGA's therapeutic effects on many diseases, including diabetes (1), cardiovascular diseases (2), cancer (3) and inflammatory bowel disease (4). However, although the biological function of CGA has been explored in detail, a paucity of reports exists on the specific mechanisms underlying the effects of CGA on inflammatory responses and mucosa damage in colitis.

Recently, studies have focused on the importance of microbiota to intestinal health, and have proven it to be critical for the maintenance of the intestinal immune system and the prevention of epithelial mucosa (5). Importantly, the microbiota is suggested to mediate the beneficial effects of dietary factors on colitis. CGA has been proven to modulate intestinal microbiota, through methods such as selectively increasing the relative abundance of the *Bifidobacterium spp*. and *Clostridium coccoides*-*Eubacterium rectale* groups in humans (6), *Akkermansia* in rodents (7), and *lactobacillus* in pigs (8). However, the mechanism by which changes in certain bacteria mediate CGA's effects on intestinal function and inflammation remains to be elucidated.

It is suggested that dietary CGA is only absorbed at a rate of approximately 33% in the small intestine, and as a result, most dietary CGA is able to reach the large intestine (9). In the present study, mice were treated with indomethacin, which induced ulceration in the small intestine and colon (10); this allowed for a focus on the effects of CGA on the colon. The changes in microbiota composition that may contribute to the beneficial effects of CGA on inflammatory response and intestinal function were identified. Through microbiota transplantation and LPS analyses, this study revealed that CGA protects against indomethacin-induced inflammation and mucosa damage by decreasing *Bacteroides*-derived LPS.

## Materials and Methods

### Animal Experiments

**Figure 1:**
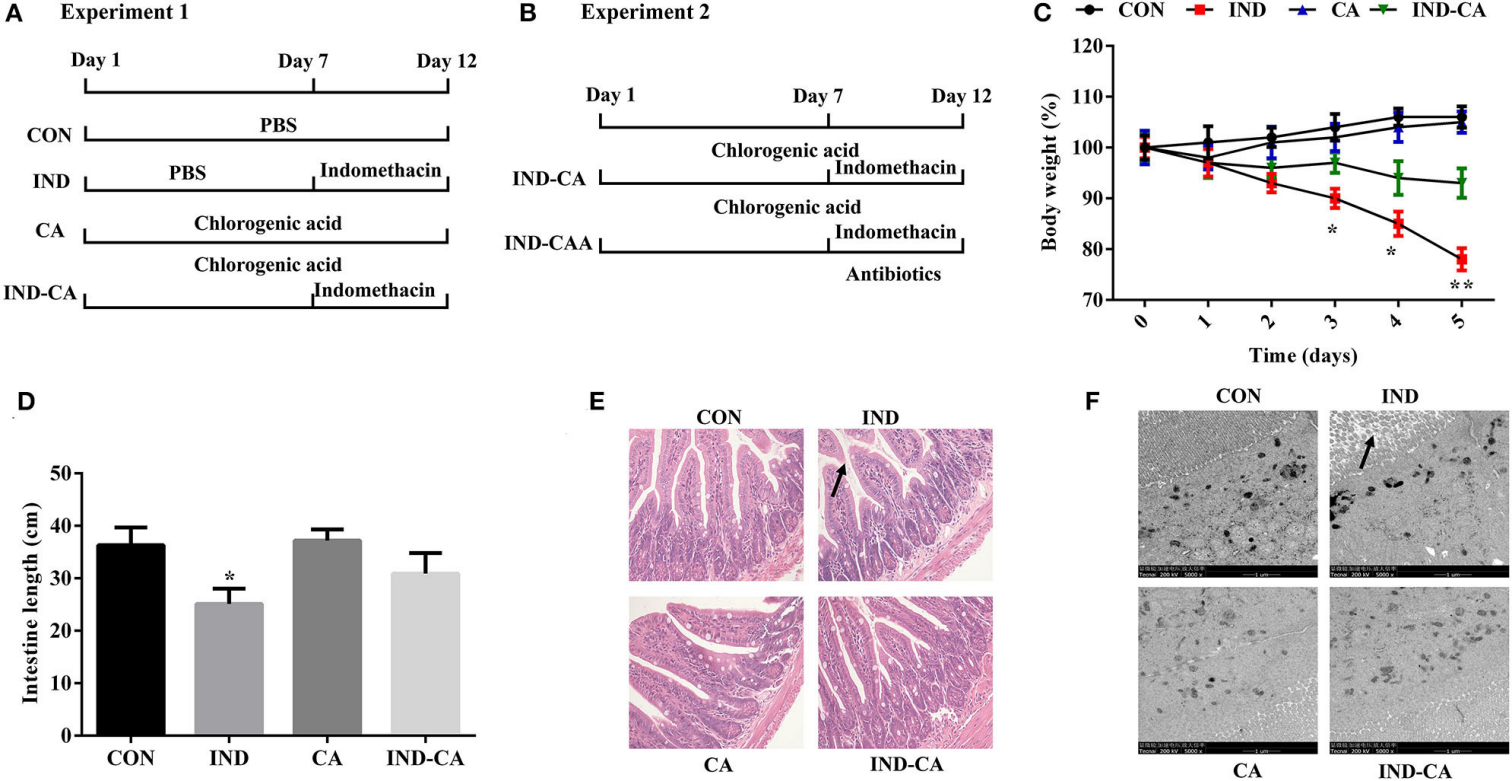
Chlorogenic acid prevented morphological damage to intestines of indomethacin-treated mice. A timeline with treatment in Experiment 1 **(A)** and Experiment 2 **(B)**; **(C)** Body weight changes after the mice were gavaged with indomethacin; **(D)** Intestinal length; **(E)** HE staining of colon morphology (200×); **(F)** Ultrastructural observation of microvilli in colon by TEM (5000×). The morphology changes were marked with black arrows in **(E,F)**. Values are expressed as mean ± SEM, *n* = 8. ^*^P < 0.05; ^**^P < 0.01.

Experiment 1: Thirty-two C57BL/6J mice (7 weeks old) were purchased from Hunan SJA Laboratory Animal Co., Ltd (Changsha, China). After acclimating for 1 week, they were each assigned to one of four treatment groups: (1) Control group (CON); (2) Indomethacin group (IND); (3) Chlorogenic acid group (CA); (4) Indomethacin and chlorogenic acid group (IND-CA) (Figure 1A). Mice in the IND group were gavaged with PBS for the first 7 days and then with 5 mg/kg indomethacin (Selleck Chemicals, Houston, TX, USA) for the last 5 days; Mice in the CA group were gavaged with 50 mg/kg chlorogenic acid (Sigma, St. Louis, MO, USA) for the whole 12-day experiment; Mice in the IND-CA group were gavaged with chlorogenic acid for the whole period and with indomethacin for the last 5 days. Body weight was recorded daily for the last 6 days. Experiment 2: Sixteen mice (7 weeks old) were allowed to acclimate for 1 week, and then assigned to either the IND-CA or IND-CAA group (*n* = 8) (Figure 1B). Mice in the IND-CA group were treated with the same protocol as in Experiment 1 with regular drinking water, while mice in the IND-CAA group were treated as did in the IND-CA group and further treated with antibiotic water (1 g/L each of gentamicin and streptomycin, and 0.5 g/L each of ampicillin and vancomycin; Meilun Bio, Dalian, China) from Day 8 and onward. All animals were maintained in plastic cages under standard conditions and had free access to feed and water.

The experimental protocol was approved by the Institutional Animal Care and Use Committee of Hunan Agricultural University (20190501) and mice were treated according to the animal care guidelines of Hunan Agricultural University (Changsha, China).

### Gut Microbiota Transplantation

After treating with the antibiotic water for 10 days, the microbiota-depleted mice were given transplants of donor microbiota. Fecal samples (100 mg) were obtained from mice in the CON, CA, and IND-CA groups. The collected fecal samples were re-suspended in 1.0 mL sterile PBS and then centrifuged at 3000 *g*. The antibiotic-treated mice were gavaged with 0.1 mL/day microbiota supernatant for 5 days. After microbiota transplantation, the mice who received feces from these three groups were designated as MT-CON, MT-CA, and MT-IND-CA, respectively. Finally, these mice were further gavaged with 5 mg/kg indomethacin for another 5 days.

### Sample Collection

At the end of the experiments, all animals were sacrificed and intestine length was measured. Then, colonic samples were collected and immediately frozen in liquid nitrogen and stored at −80°C until analysis of gene and protein expression was conducted. Additionally, colonic samples were either fixed in 10% neutral formaldehyde or in 2.5% glutaraldehyde for histological examination. Finally, cecum contents were collected for the analysis of microbiota composition.

### Histological Analyses

The samples (1.0–1.5 cm proximal to the anus) fixed in formaldehyde were embedded in paraffin and 8-μm sections were stained with hematoxylin and eosin (HE) for the observation of histological morphology as previously described (11). In addition, pre-treatment of colonic samples and transmission electron microscopy (TEM) were performed. Briefly, the samples fixed in glutaraldehyde were rinsed in PBS twice and then post-fixed in osmium tetroxide. Then, the samples were dehydrated in 30, 50, 70, 90, and 100% acetones solutions, respectively. Finally, the dehydrated samples were embedded in araldite, and then stained with uranyl acetate and lead citrate for observation under a transmission electron microscope (Zeiss, Thornwood, NY, United States).

### RT-qPCR Analysis

RT-qPCR analysis was performed as previously described (12). Briefly, a TRIZOL kit (Takara, Dalian, China) was used for the extraction of total RNA from colonic samples (1.5–2.0 cm proximal to the anus), which was reverse transcribed using a cDNA Reverse Transcription Kit (Takara). Then, RT-qPCR was performed with SYBR Green mix (Takara) according to the manufacturer's instructions. The primers are listed in Table 1.

**Table 1:** Primer sequences for RT-qPCR.

**Gene**	**5′-3′ Primer sequence**
TNFα	F: ATGAGAAGTTCCCAAATGGC
	R: CTCCACTTGGTGGTTTGCTA
IL1β	F: TGCCACCTTTTGACAGTGATG
	R: AAGGTCCACGGGAAAGACAC
IL6	F: CCTCTCTGCAAGAGACTTCCAT
	R: AGTCTCCTCTCCGGACTTGT
IL8	F: CTAGGCATCTTCGTCCGTCC
	R: CAGAAGCTTCATTGCCGGTG
β-actin	F: TGTCCACCTTCCAGCAGATGT
	R: AGCTCAGTAACAGTCCGCCTAGA

### Protein Qualification by the Wes Simple Western System

Protein qualification was performed using the Wes Simple Western System (ProteinSimple, San Jose, CA, USA) as previously described (13). Proteins extracted from colonic samples (2.0–3.0 cm proximal to the anus) were mixed with dithiothreitol, Master Mix, Simple Western Sample Buffer, and fluorescent standards (ProteinSimple) and loaded into Wes 25-well plates. Thereafter, primary antibodies (phosphorylated NFκB (pNFκB) and NFκB, Abcam, Cambridge, MA, USA), secondary antibodies, stacking gel matrix, luminol-peroxide mixture, and separation gel matrix were added. Results were collected using the “gel view” function of the Protein Simple software.

### Immunofluorescence Assay for Tight Junction Proteins

Colonic samples embedded in paraffin were sliced into 8-μm sections and incubated with primary antibodies (occludin and claudin-1) overnight at 4°C. Subsequently, the slices were further incubated with the secondary antibodies at 37°C for 1 h. They were then mounted on a slide with DAPI (Sigma) and representative pictures were taken using a Zeiss LSM880 confocal microscope (Shanghai, China).

### FITC-Dextran Intestinal Permeability Assay

Oral gavage of FITC-dextran (Sigma) was performed to determine intestinal permeability. Animals were gavaged with 600 mg/kg FITC-dextran. Serum samples were collected 4 h later, and FITC-dextran concentrations were assayed by fluorometry.

### Determination of Myeloperoxidase, Eosinophil Peroxidase and LPS Contents

Myeloperoxidase (MPO), eosinophil peroxidase (EPO), and LPS concentrations were determined using ELISA quantitative kits (Meimian, Nanjing, China) according to the manufacturer's instructions.

### Gut Microbiota Profiling

Samples of cecum contents were collected and homogenized, and then used for DNA extraction using the QIAamp DNA stool Mini Kit (Qiagen, Shanghai, China). The V3–V4 regions of bacterial 16S rRNA gene sequences were amplified. PCR was performed in a solution consisting of 12.5 mL of Phusion High-Fidelity PCR Master Mix (Beverly, MA, United States), 50 ng of template DNA, and 1 mL of forward and reverse primer, respectively. The PCR products (400–450 bp) were purified for MiSeq Illumina sequencing, which was performed using the Illumina HiSeq2500 platform (San Diego, CA, United States). Then, the merged reads were assigned to each sample based on their unique barcodes. Based on 97% sequence similarity, OTUs were obtained by clustering the high-quality clean tags. Analysis was performed with representative OTUs using the Greengenes database with the RDP algorithm.

### Statistical Analysis

Data were collected for statistical analysis performed using the *t*-test or one-way ANOVA with SPSS 18.0 (Chicago, IL, USA). All data were expressed as means ± standard error (SEM) and a *P*-value of <0.05 was considered statistically significant.

## Results

### Chlorogenic Acid Prevented Morphological Damage to Intestines of Indomethacin-Treated Mice

The indomethacin-treated group showed significantly decreased body weight at the third day of treatment, while no changes were observed in the mice of the other three groups (Figure 1C). Intestinal length was significantly decreased in the indomethacin-treated group, while this change was prevented when chlorogenic acid was added to the treatment protocol (Figure 1D). HE staining results showed partial loss and sloughing of colonic villi in mice treated with indomethacin, while no changes were observed in the villi shapes of the other three groups (Figure 1E). Moreover, TEM results showed that the microvilli were irregular as a result of indomethacin treatment, while no such change was observed in the other groups (Figure 1F).

### Chlorogenic Acid Prevented Inflammation in the Colons of Indomethacin-Treated Mice

**Figure 2:**
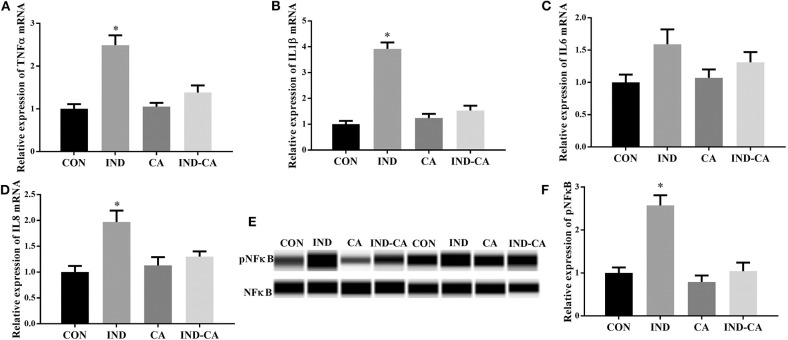
Chlorogenic acid prevented inflammation in the colons of indomethacin-treated mice. Relative mRNA expression of TNFα **(A)**, IL1β **(B)**, IL6 **(C)**, and IL8 **(D)**; **(E)** pNFκB expression determined by the WES Simple Western System. A full image of panel **(E)** shown in Supplementary Figure 1; **(F)** Relative abundance of pNFκB, **(F)** is the quantification of the WB in E. Values are expressed as mean ± SEM, *n* = 8 for results of gene expression and *n* = 3 for results of protein expression. ^*^P < 0.05.

As shown in Figure 2, indomethacin treatment gave rise to significant increases in the gene expression of TNFα, IL1β, and IL8 in the colon, while chlorogenic acid prevented these increases. No significant change in the gene expression of IL6 was observed among the four treatment groups. In addition, pNFκB protein expression in the colon was significantly increased due to indomethacin treatment, while no change was observed in the mice treated with chlorogenic acid.

### Chlorogenic Acid Prevented Intestinal Barrier Dysfunction in Indomethacin-Treated Mice

**Figure 3:**
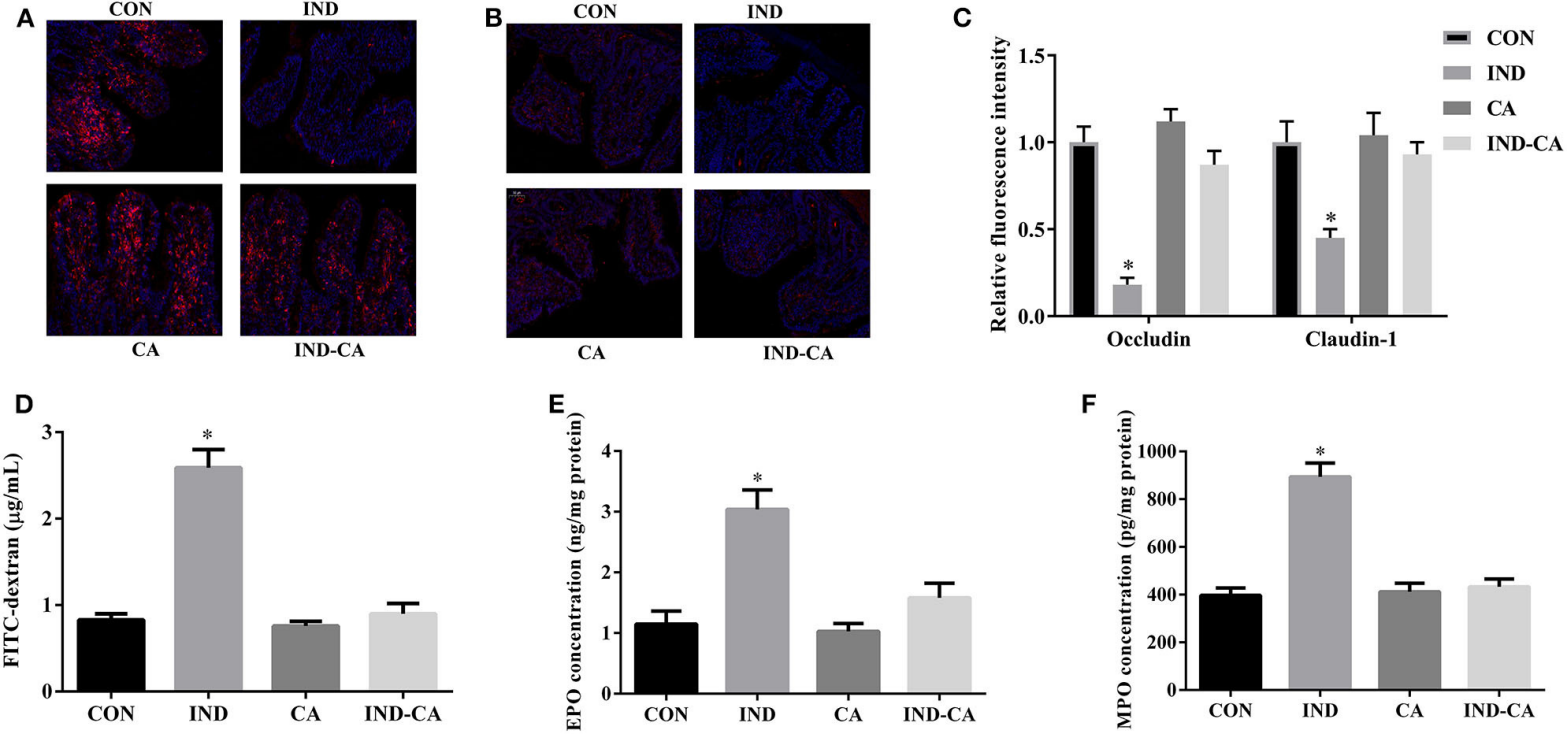
Chlorogenic acid prevented intestinal barrier dysfunction in indomethacin-treated mice. Occludin **(A)** and claudin-1 **(B)** expression in colon by immunofluorescence assay (Red, tight junction proteins; Blue, DAPI); **(C)** Relative fluorescence intensity of occluding and claudin-1 (*n* = 3); **(D)** Concentration of FITC-dextran in serum; EPO **(E)** and MPO **(F)** concentrations in colonic tissue. Values are expressed as mean ± SEM, *n* = 8. ^*^P < 0.05.

Indomethacin treatment significantly decreased occludin and claudin-1 protein expression, while it failed to cause such changes in mice treated with chlorogenic acid (Figures 3A–C). The FITC fluorescence intensity and the EPO and MPO concentrations were significantly increased by indomethacin treatment, while no such changes were observed in mice treated with chlorogenic acid (Figures 3D–F).

### Chlorogenic Acid Decreased *Bacteroides* Abundance and LPS Content in Indomethacin-Treated Mice

**Figure 4:**
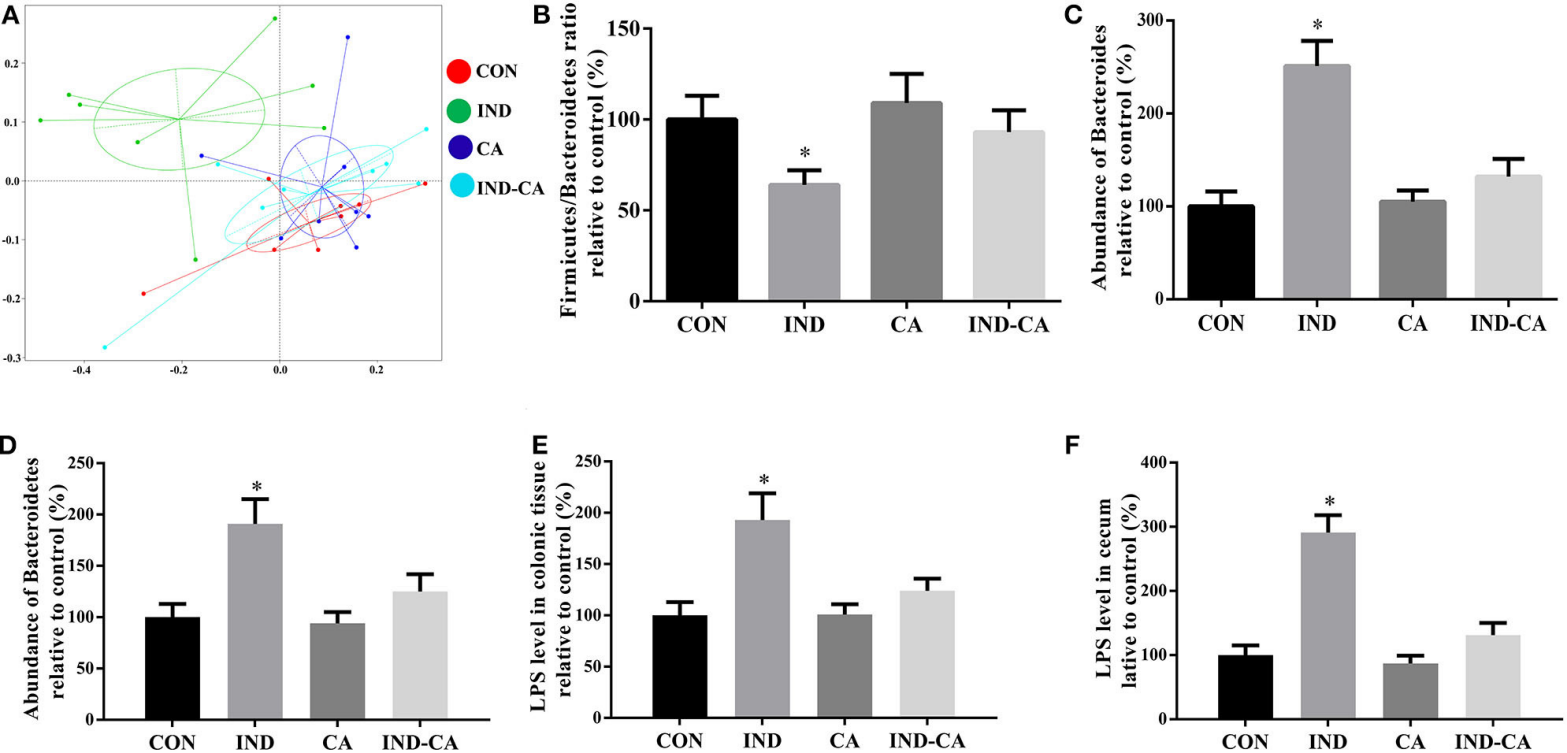
Chlorogenic acid decreased *Bacteroides*-derived LPS content in indomethacin-treated mice. **(A)** PCoA plot of the cecum microbiota; **(B)** Relative ratio of *Firmicutes* to *Bacteroidetes*; Relative abundance of *Bacteroides* at the genus level **(C)** and *Bacteroidetes* at the phylum level **(D)**; Relative LPS content in the colonic tissue **(E)** and cecum **(F)**. Values are expressed as mean ± SEM, *n* = 8. ^*^P < 0.05.

The microbiota of the ceca of different groups were analyzed. PCoA analysis highlighted the differences in the composition of microbiota between the IND group and the other three groups (Figure 4A). The *Firmicutes*/*Bacteroidetes* ratio was significantly lower in the IND group when compared with the ratios in the other three groups (Figure 4B). Additionally, the relative abundance of *Bacteroides* at the genus level and *Bacteroidetes* at the phylum level in the microbiota of the IND group was significantly higher than those of the other three groups (Figures 4C,D). Notably, LPS levels in the colonic tissue and cecum had significantly increased in the IND group, while no similar change was observed in the other three groups (Figures 4E,F).

### Microbiota Depletion by Antibiotics Blocked the Anti-inflammatory Effect of Chlorogenic Acid in Indomethacin-Treated Mice

**Figure 5:**
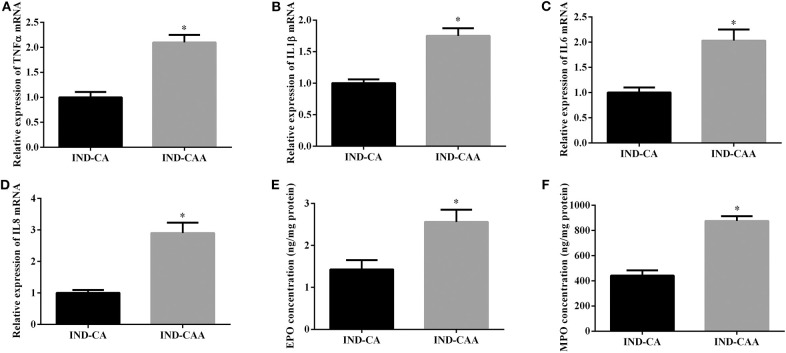
Microbiota depletion by antibiotics blocked the anti-inflammatory effect of chlorogenic acid in indomethacin-treated mice. Relative mRNA expression of TNFα **(A)**, IL1β **(B)**, IL6 **(C)**, and IL8 **(D)**; EPO **(E)** and MPO **(F)** concentrations in colonic tissue. Values are expressed as mean ± SEM, *n* = 8. ^*^P < 0.05.

As shown in Figure 5, mice in the IND-CAA group had higher gene expression of TNFα, IL1β, IL6, and IL8 in colonic tissue than those in the IND-CA group. Additionally, mice in the IND-CAA group had higher concentrations of EPO and MPO in colonic tissue than did those in the IND-CA group.

### CGA-Originated Microbiota Prevented Intestinal Morphology Damage and Barrier Dysfunction Caused by Indomethacin

**Figure 6:**
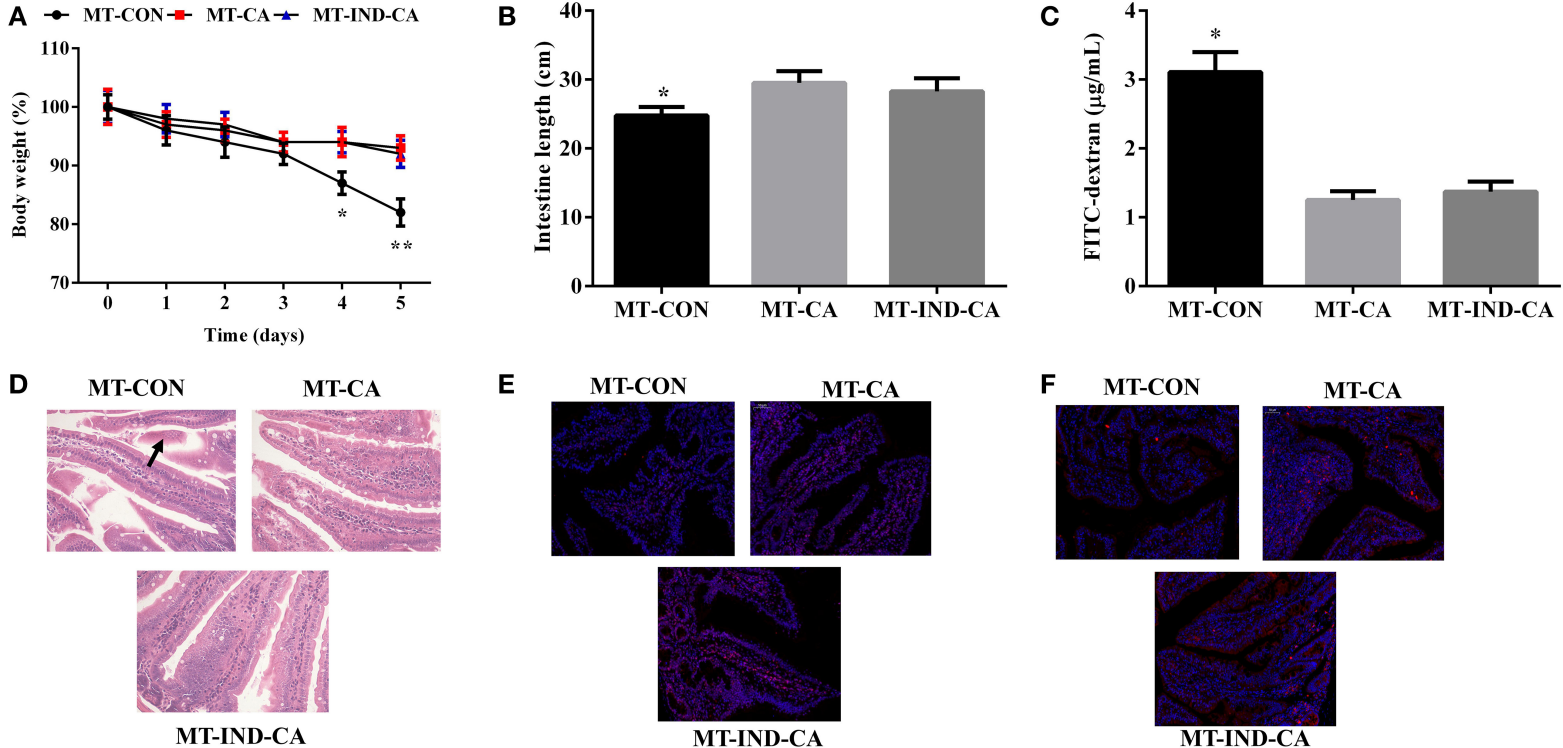
CGA-originated microbiota prevented intestinal morphology damage and barrier dysfunction caused by indomethacin. **(A)** Body weight changes after the mice were gavaged with indomethacin; **(B)** intestinal length; **(C)** Concentration of FITC-dextran in serum; **(D)** HE staining of colon morphology (200×), The morphology changes were marked with black arrows; Occludin **(E)** and claudin-1 **(F)** expression in colon by immunofluorescence assay (Red, tight junction proteins; Blue, DAPI). Values are expressed as mean ± SEM, *n* = 4. ^*^P < 0.05; ^**^P < 0.01.

After the gut microbiota transplantation program, mice in the MT-CA and MT-IND-CA groups experienced less body weight loss than mice in the MT-CON group (Figure 6A). Compared with those in MT-CON group, mice in MT-CA and MT-IND-CA exhibited longer intestine length and lower FITC fluorescence intensity in serum (Figures 6B,C). HE staining results showed that mice in the MT-CON group had partial loss and sloughing of colonic villi, while no such changes were observed in mice of the MT-CA and MT-IND-CA groups (Figure 6D). In addition, results of an immunofluorescence assay showed that mice in the MT-CON group had a lower expression level of occludin and claudin-1 than did mice in MT-CA and MT-IND-CA (Figures 6E,F).

### CGA-Originated Microbiota Prevented Intestinal Inflammation and Inhibited the Growth of LPS-Producing *Bacteroides* Caused by Indomethacin

**Figure 7:**
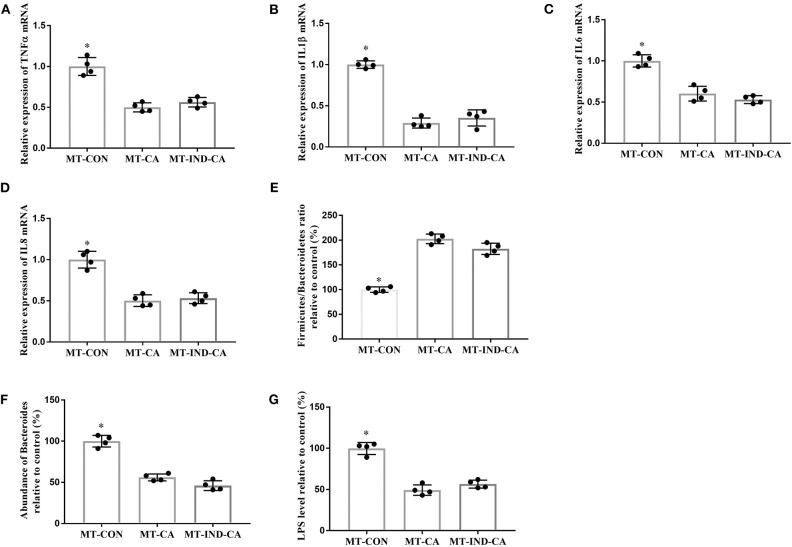
CGA-originated microbiota prevented intestinal inflammation and inhibited the growth of LPS-producing *Bacteroides* caused by indomethacin. Relative mRNA expression of TNFα **(A)**, IL1β **(B)**, IL6 **(C)**, and IL8 **(D)**; **(E)** Relative ratio of *Firmicutes* to *Bacteroidetes*; **(F)** Relative abundance of *Bacteroides* at the genus level; **(G)** Relative LPS content in the colonic tissue. Values are expressed as mean ± SEM, *n* = 4. ^*^P < 0.05.

Mice in the MT-CON group had higher gene expression of TNFα, IL1β, IL6, and IL8 in colonic tissue than those in the MT-CA and MT-IND-CA groups (Figures 7A–D). Additionally, mice in the MT-CON group had a lower *Firmicutes*/*Bacteroidetes* ratio and more abundant *Bacteroides* and LPS levels when compared with those in the MT-CA and MT-IND-CA groups (Figures 7E–G).

## Discussion

The natural polyphenol CGA has numerous biological functions, and the protective effects of CGA on the gastrointestinal tract have been widely demonstrated in a variety of *in vivo* and *in vitro* models (4, 14, 15). Dietary CGA plays important roles in the maintenance of intestinal morphology, intestinal permeability, and barrier function via different mechanisms. Since intestinal permeability is positively associated with mitochondrial dysfunction, a previous study has suggested that CGA exerts its beneficial effects by decreasing mitochondrial lipid peroxidation and ameliorating mitochondrial respiratory chain dysfunction (15). The strength of the intestinal physical barrier is determined by its major structural basis, the tight junction. Moreover, the activation of myosin light chain kinase (MCK) is essential for intestinal barrier function, as it triggers changes in tight junctions by promoting the phosphorylation of myosin light chain. CGA has been proven to decrease MCK expression and improve the dynamic distribution of tight junction proteins in a rodent model of colitis (16), confirming its beneficial effects on intestinal barrier function. This study found that CGA prevents increases in intestinal leakage, as serum FITC-labeled dextran was found to decrease. Moreover, CGA increases the expression of tight junction proteins in the colon. These results suggest that CGA exerts the same effects on intestinal permeability and barrier function in indomethacin-induced colitis, which gives insight into the protective effects of CGA on the gastrointestinal tract in different models of colitis. Ulcerative colitis is a common form of inflammatory bowel disease (IBD) characterized by inflammation and colonic mucosa. It is important to alleviate the inflammatory response during IBD treatment. *In vitro* experiments have shown that CGA exerts strong anti-inflammatory effects via the activation of the Nrf2 or NFκB signaling pathway in Caco-2 cells challenged with a mixture of phorbol, 12-myristate, 13-acetate, and interferon γ or a mixture of TNFα and H_2_O_2_ (4, 14). Moreover, CGA prevents inflammatory cell infiltration and inhibits the over-accumulation of inflammatory cytokines in the rodent model of colitis induced by dextran sulfate sodium (4, 7). However, although the anti-inflammatory effects of CGA have been proven in this model of IBD, further evidence is needed to confirm these findings. This study found that CGA deceases the expression of inflammatory cytokines and inhibits the expression of NFκB in indomethacin-induced colitis, and these new findings allow for a broader understanding of the effects of CGA on the prevention of IBD.

Indomethacin, one of the most widely used non-steroidal anti-inflammatory drugs, exerts adverse effects on gastrointestinal mucosa (17). Indomethacin treatment results in extraintestinal lesions, inflammatory responses, and the inhibition of mucosal cell renewal. Consequently, it is a priority to find strategies to effectively and safely prevent gastrointestinal mucosal damage and inflammation in patients treated with indomethacin. Additionally, based on its adverse effects on intestine, indomethacin has been used to induce colonic ulceration in rodents, which has been suggested to be an appropriate animal model for IBD research (10). The model has some advantages, such as being easily induced and sharing some similarities with Crohn's disease. However, the detailed mechanism underlying the pathogenesis of indomethacin-induced colonic ulceration and inflammation remains to be elucidated. This study provides evidence that indomethacin treatment damages intestinal epithelial cell barrier integrity, increases gene expression of pro-inflammatory cytokines, increases protein expression of pNFκB, and affects microbiota composition. It is possible that indomethacin might affect the intestinal microflora and damage the epithelium, leading to the activation of inflammatory signaling pathways such as NFκB, which causes pro-inflammatory cytokines to be over-expressed and accumulate in the intestine. CGA might directly prevent indomethacin-induced activation of NFκB and its down-stream targets including certain cytokines, as numerous previous studies have demonstrated in certain rodent models of colitis (4, 7). Another possibility is that CGA could also indirectly affect the NFκB pathway via inhibition of certain upstream signals, such as changes in microbiota composition and related metabolites (7, 18).

Several studies have demonstrated the influence of CGA on gut microbiota composition. A previous study found that CGA increases the abundance of the phyla *Firmicutes* and *Bacteroidetes* in weaned piglets (19). The researchers suggested that the increased abundance of *Bacteroidetes*, which produces short-chain fatty acids (SCFAs) such as butyrate, would partially benefit intestinal function. Surprisingly, CGA decreased the abundance of the genus *Bacteroides* at the same time. The effects of CGA on the concentration of SCFAs align with another study on weaned piglets, which found that dietary CGA supplementation led to an increase in butyric acid in the intestine (8). However, a study on growing pigs did not show increased abundance of *Firmicutes* and *Bacteroidetes* (20). Unfortunately, the content of SCFAs was not determined, but they found that other metabolites such as 5-hydroxytryptamine increased, accompanied by changes in the microbiota composition of the colon. The effects of CGA on experimental colitis induced by dextran sulfate sodium were explored by another study, and microbiota was suggested to play a critical role in mediating the ameliorative effects of CGA (7). The proportions of *Firmicutes* and *Bacteroidetes* are decreased in colitis mice. Notably, a significantly increased proportion of *Akkermansia*, a mucin-degrading bacterium, is suggested to improve the mucus layer integrity. We found that dietary CGA increases the *Firmicutes*/*Bacteroidetes* ratio in indomethacin-treated mice, which is in accordance with the aforementioned results in several animal models of intestinal dysfunction. However, unlike previous studies, this study found that CGA affects *Bacteroides* abundance and decreases LPS content by decreasing the level of LPS-producing *Bacteroides* in the colon. The *Bacteroides* genus is sometimes associated with health conditions (21); some of its strains such as *Bacteroides dorei* and *Bacteroides fragilis* are suggested to promote the development of diseases such as diabetes and coeliac disease (22, 23). LPS is one of the major products of *Bacteroides* and may induce intestinal epithelial barrier dysfunction and inflammation (24). Notably, CGA had little effect on indomethacin-induced inflammation in the colons of antibiotic-treated mice, which suggests the prevention of intestinal inflammation by CGA could be largely influenced by the microbiota. Importantly, mice colonized with fecal microbiota from mice in the CA and CA-IND groups showed similar effects (intestine length, morphology and integrity, cecum *Bacteroides* abundance and LPS content, and colonic inflammation) as CGA did after indomethacin treatment. It can be inferred from these facts that the intestinal microbiota, primarily LPS-producing *Bacteroides*, mediate the effects of CGA on the prevention of indomethacin-induced inflammation.

In conclusion, it was found that CGA could protect against indomethacin-induced inflammation and mucosa damage. Importantly, the results also suggest that CGA may protect intestine integrity and alleviate inflammatory responses, primarily by inhibiting the growth of *Bacteroides* and the accumulation of *Bacteroides*-derived LPS. This newly identified mechanism broadens the base of knowledge on how CGA exerts its protective effects on intestinal damage, and may help to provide strategies for the prevention of gastrointestinal mucosal damage in patients treated with indomethacin.

## Data Availability Statement

The raw data supporting the conclusion of this article will be made available by the authors, without undue reservation, to any qualified researchers.

## Ethics Statement

The animal study was reviewed and approved by Institutional Animal Care and Use Committee of Hunan Agriculture University.

## Author Contributions

YY and HY designed the experiment and drafted the manuscript. YY, XZ, and KG carried out the animal trials and sample analysis. FZ did data analysis work. HY was responsible for the integrity of the work as a whole. All authors reviewed and approved the final manuscript.

## Conflict of Interest

The authors declare that the research was conducted in the absence of any commercial or financial relationships that could be construed as a potential conflict of interest.

## Supplementary Material

Supplementary Figure 1 A full image of Figure 2E.
