# Supplementary figures and images for: Chlorogenic Acid Protects Against Indomethacin-Induced Inflammation and Mucosa Damage by Decreasing Bacteroides-Derived LPS
Source: Front Immunol. 2020 Jun 3;11:1125. doi: 10.3389/fimmu.2020.01125 (PMC7283755; doi:10.3389/fimmu.2020.01125)

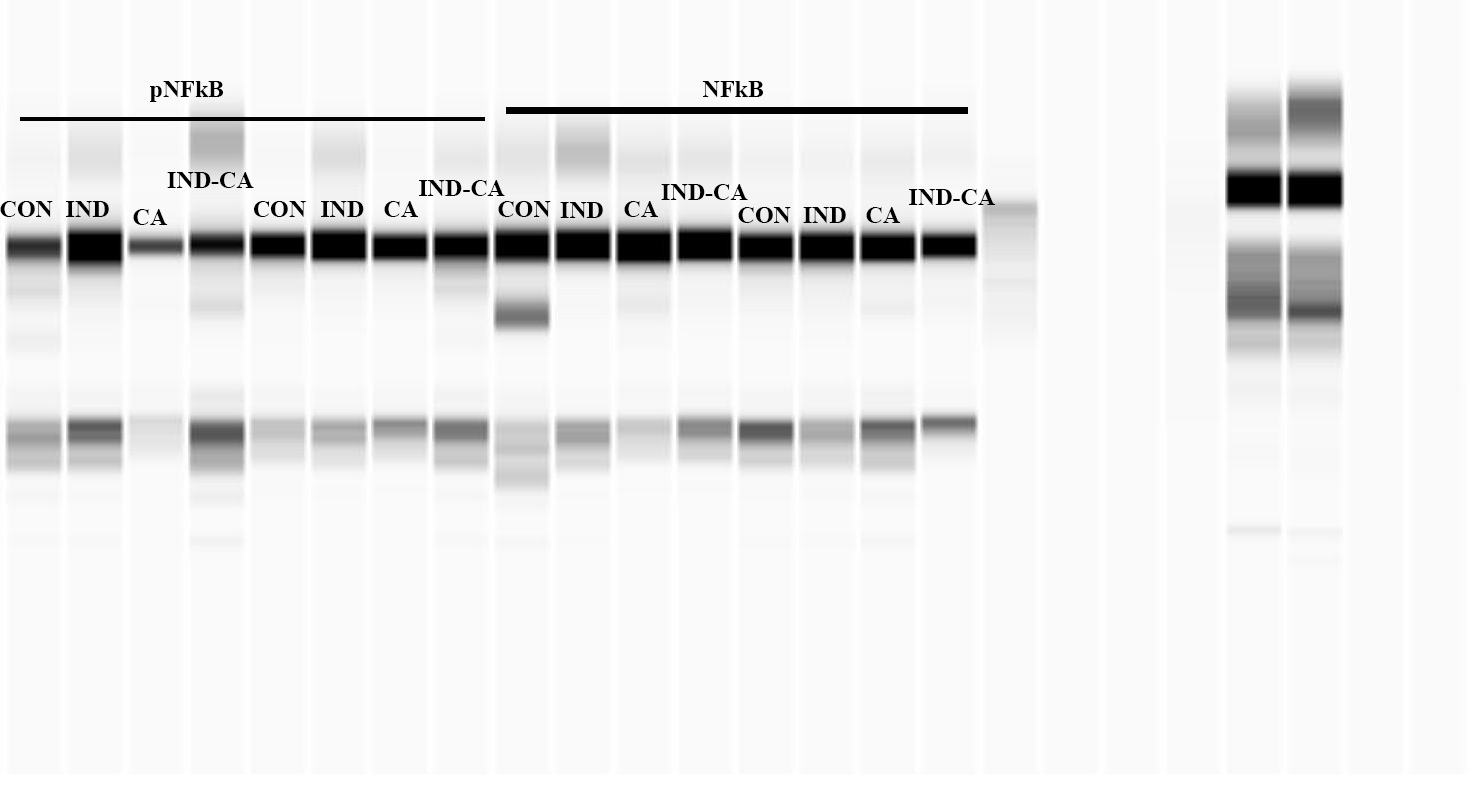

Supplement: Supplementary Figure 1 — A full image of Figure 2E. [file Image_1.TIF]
